# Plasma SARS-CoV-2 nucleocapsid antigen levels are associated with progression to severe disease in hospitalized COVID-19

**DOI:** 10.1186/s13054-022-04153-3

**Published:** 2022-09-14

**Authors:** Katherine D. Wick, Aleksandra Leligdowicz, Andrew Willmore, Sidney A. Carrillo, Rajani Ghale, Alejandra Jauregui, Suzanna S. Chak, Viet Nguyen, Deanna Lee, Chayse Jones, Robin Dewar, H. Clifford Lane, Kirsten N. Kangelaris, Carolyn M. Hendrickson, Kathleen D. Liu, Pratik Sinha, David J. Erle, Charles R. Langelier, Matthew F. Krummell, Prescott G. Woodruff, Carolyn S. Calfee, Michael A. Matthay, the COMET Consortium, Yumiko Abe-Jones, Alexander Beagle, Sharvari Bhide, Gabriela K. Fragiadakis, Ana Gonzalez, Omid Jamdar, Norman Jones, Tasha Lea, Carolyn Leroux, Jeff Milush, Logan Pierce, Priya Prasad, Sadeed Rashid, Nicklaus Rodriguez, Austin Sigman, Alyssa Ward, Michael Wilson

**Affiliations:** 1 Cardiovascular Research Institute, University of California San Francisco, 503 Parnassus Ave, HSE 760, San Francisco, CA 94143 USA; 2 Division of Critical Care, Departments of Medicine and Microbiology and Immunology, Western University, London, ON Canada; 3 Robarts Research Institute, Western University, London, ON Canada; 4 Division of Pulmonary and Critical Care Medicine, Department of Medicine, University of California San Francisco, San Francisco, CA USA; 5 Division of Pulmonary and Critical Care Medicine, Department of Medicine, Zuckerberg San Francisco General Hospital and Trauma Center, University of California San Francisco, San Francisco, USA; 6 Virus Isolation and Serology Laboratory, Applied and Developmental Directorate, Frederick National Laboratory, Frederick, MD USA; 7 Division of Clinical Research, National Institute of Allergy and Infectious Diseases, National Institutes of Health, Bethesda, MD USA; 8 Department of Hospital Medicine, University of California San Francisco, San Francisco, CA USA; 9 Division of Nephrology, Department of Medicine, University of California San Francisco School of Medicine, San Francisco, CA USA; 10 Division of Critical Care Medicine, Department of Anesthesia, University of California San Francisco School of Medicine, San Francisco, CA USA; 11 Department of Anesthesia, Division of Critical Care, Washington University, St. Louis, MO USA; 12 Division of Clinical and Translational Research, Washington University School of Medicine, St. Louis, MO USA; 13 Lung Biology Center, University of California San Francisco, San Francisco, CA USA; 14 ImmunoX Initiative, University of California San Francisco, San Francisco, CA USA; 15 UCSF CoLabs, University of California San Francisco, San Francisco, CA USA; 16 Division of Infectious Diseases, University of California San Francisco, San Francisco, CA USA; 17 Chan Zuckerberg Biohub, University of California San Francisco, San Francisco, CA USA; 18 Departments of Medicine and Laboratory Medicine, University of California San Francisco, San Francisco, CA USA; 19 Departments of Medicine and Anesthesia, University of California San Francisco, San Francisco, CA USA

## Abstract

**Background:**

Studies quantifying SARS-CoV-2 have focused on upper respiratory tract or plasma viral RNA with inconsistent association with clinical outcomes. The association between plasma viral antigen levels and clinical outcomes has not been previously studied. Our aim was to investigate the relationship between plasma SARS-CoV-2 nucleocapsid antigen (N-antigen) concentration and both markers of host response and clinical outcomes.

**Methods:**

SARS-CoV-2 N-antigen concentrations were measured in the first study plasma sample (D0), collected within 72 h of hospital admission, from 256 subjects admitted between March 2020 and August 2021 in a prospective observational cohort of hospitalized patients with COVID-19. The rank correlations between plasma N-antigen and plasma biomarkers of tissue damage, coagulation, and inflammation were assessed. Multiple ordinal regression was used to test the association between enrollment N-antigen plasma concentration and the primary outcome of clinical deterioration at one week as measured by a modified World Health Organization (WHO) ordinal scale. Multiple logistic regression was used to test the association between enrollment plasma N-antigen concentration and the secondary outcomes of ICU admission, mechanical ventilation at 28 days, and death at 28 days. The prognostic discrimination of an externally derived “high antigen” cutoff of N-antigen ≥ 1000 pg/mL was also tested.

**Results:**

N-antigen on D0 was detectable in 84% of study participants. Plasma N-antigen levels significantly correlated with RAGE (*r* = 0.61), IL-10 (*r* = 0.59), and IP-10 (*r* = 0.59, adjusted *p* = 0.01 for all correlations). For the primary outcome of clinical status at one week, each 500 pg/mL increase in plasma N-antigen level was associated with an adjusted OR of 1.05 (95% CI 1.03–1.08) for worse WHO ordinal status. D0 plasma N-antigen ≥ 1000 pg/mL was 77% sensitive and 59% specific (AUROC 0.68) with a positive predictive value of 23% and a negative predictive value of 93% for a worse WHO ordinal scale at day 7 compared to baseline. D0 N-antigen concentration was independently associated with ICU admission and 28-day mechanical ventilation, but not with death at 28 days.

**Conclusions:**

Plasma N-antigen levels are readily measured and provide important insight into the pathogenesis and prognosis of COVID-19. The measurement of N-antigen levels early in-hospital course may improve risk stratification, especially for identifying patients who are unlikely to progress to severe disease.

**Supplementary Information:**

The online version contains supplementary material available at 10.1186/s13054-022-04153-3.

## Background

Plasma biomarkers of inflammation, organ and tissue damage, and coagulation have been investigated as pathogenic and prognostic biomarkers in COVID-19 disease [1–6]. Although these biomarkers provide insight into the mechanisms of virally mediated cell and tissue damage and the host response to SARS-CoV-2, they do not provide a direct measure of viral burden. It can be challenging to understand the importance of non-viral plasma biomarkers in relation to viral infection without concurrent quantification of viral RNA or protein.

Viral burden in SARS-CoV-2 infection is most often quantified by measurement of viral RNA or proteins in the upper respiratory tract. Higher viral load in the upper respiratory tract portends worse outcomes in some studies [7–10], while others have demonstrated no independent association [11]. Viral load in the upper airways is often not strongly associated with viral products in the plasma [12–15]. It is plausible that plasma viral burden may provide additional value. Previous studies have established that the detection of circulating SARS-CoV-2 viral RNA is associated with baseline disease severity and with clinical outcomes [12, 16–21].

Viral RNA in plasma is also associated with biomarkers of inflammation and tissue damage [12, 19, 20, 22, 23]. Circulating SARS-CoV-2 RNA is often not detectable, however, especially in early or non-critical COVID-19 [17, 24–26]. There is increasing interest in the utility of the detection of viral protein antigens in the blood, which are detectable within 2 weeks of symptom onset [27–30] and are quantifiable even in patients with mild or minimal symptoms [28–31]. However, there are currently limited studies of how SARS-CoV-2 viral antigen detection in the blood is related to disease severity and outcomes [32–37].

The aims of this study were to investigate the relationship between SARS-CoV-2 nucleocapsid antigen (N-antigen) levels detected in plasma at study enrollment (D0) and (1) biomarkers of tissue damage and host response, to COVID-19 and (2) both short-term clinical deterioration and longer-term outcomes of mechanical ventilation and death up to 28 days. We hypothesized that D0 plasma N-antigen concentration would be associated with biomarkers of inflammation, tissue damage, and coagulation and with disease severity.

## Methods

### Study design

The COVID-19 Multi-phenotyping for Effective Therapies (COMET) study is a prospectively enrolled observational cohort of patients with confirmed or suspected COVID-19 from three hospitals in San Francisco, CA. Sixty subjects were co-enrolled in the Immunophenotyping Assessment in a COVID-19 Cohort (IMPACC) study, and the two study protocols were aligned [38]. Full inclusion and exclusion criteria are provided in the online supplement. Briefly, hospitalized patients 18 years or older with confirmed or suspected COVID-19 infection were eligible for enrollment in COMET within 72 h of hospital admission. Plasma was collected at the time of enrollment (D0) and on study days 4, 7, and 14 during hospitalization. Additional inclusion criteria for the present study were PCR-confirmed COVID-19 infection and sufficient plasma volume to measure viral antigen concentration. Exclusion criteria were planned comfort care at the time of admission, known pregnancy, or incarceration. The primary clinical outcome was clinical status on day 7 as measured by the World Health Organization (WHO) ordinal scale (detailed in Additional file 1). Secondary outcomes were any intensive care unit (ICU) admission for participants not admitted to the ICU at enrollment, invasive mechanical ventilation (MV) on day 28, and death on day 28. Informed consent was obtained from study participants or a designated surrogate decision-maker when available. The Institutional Review Board granted a waiver of consent for 15 study participants from whom direct consent could not be obtained because of death during the study or inability to contact the participant or their surrogate after three separate attempts. This study was approved by the UCSF Institutional Review Board, IRB 20-30497 and the COMET and IMPACC scientific leadership committees.

### Sample collection and measurements

EDTA-anticoagulated blood collected at study enrollment (within 72 h of hospital admission) was centrifuged at an ambient temperature at 1000 g for 10 min, and plasma was collected and stored at − 80 °C. SARS-CoV-2 viral nucleocapsid protein (N-antigen) levels were measured in plasma using a single-molecule immune bead assay (Quanterix, Billerica, MA, USA). N-antigen concentrations below the lower limit of detection of 3 pg/mL were assigned a value of 2.9 pg/mL. Plasma interleukin (IL)-6, IL-8, IL-18, IL-10, interferon-gamma induced protein (IP)-10, surfactant protein D (SPD), receptor for advanced glycation end-products (RAGE), angiopoietin-2 (Ang-2), and soluble tumor necrosis factor receptor (sTNFR)-1 were measured using multiplex magnetic bead immunoassays (Luminex, R&D Systems, Minneapolis, MN, USA), and Protein C was measured by ELISA (Helena Laboratories, Beaumont, TX).

### Statistical analysis

Continuous variables are presented as mean (SD) or median (IQR) and compared by unpaired *t* test if normally distributed or by the Wilcoxon rank-sum test if not normally distributed. Categorical variables are presented as n (percent) and compared by chi-square or Fisher’s exact test. Spearman rank correlation was used to test the relationship between plasma N-antigen concentrations and other plasma biomarkers, with a Bonferroni correction for multiple comparisons.

To assess the relationship between N-antigen concentration per 500 pg/mL increase and clinical status at one week, a proportional odds model was fit with a modified ordinal scale as the dependent variable. Categories on the original 8-point WHO ordinal scale with few observations (less than approximately 5% of the sample) at one week were collapsed for the ordinal regression, resulting in a 5-category ordinal scale (Additional file 1: Table S1) ranging from 1 (ambulatory) to 5 (mechanically ventilated or deceased). Separate logistic regression models were fit to assess the relationship between N-antigen concentration per 500 pg/mL increase and ICU admission among study participants not admitted to the ICU at D0 and 28-day mechanical ventilation (MV) or death. Adjustment variables were selected if they were determined pre-hoc to be associated with COVID severity or outcomes, differed substantially across viral antigen quartiles, and/or were likely to affect viral load. All models were adjusted for age, sex, body mass index (BMI), race (white/non-white), symptom duration (days), presence of diabetes, presence of hypertension, immunosuppression (defined as > 4 weeks of 20 mg prednisone equivalent, other immunosuppressive medication, HIV diagnosis, or bone marrow/solid organ transplant), smoking status (current smoker yes/no), remdesivir treatment, steroid treatment for more than one day and within one week before until 21 days after enrollment, enrollment date, and 8-point WHO ordinal scale at D0. Models for clinical status at one week and ICU admission were adjusted for vaccination status. Models for 28-day mechanical ventilation and death were not adjusted for vaccine status as no vaccinated participants were deceased or mechanically ventilated at 28 days.

We evaluated the prognostic value of an externally defined single N-antigen cutoff of ≥ 1000 pg/mL for clinical deterioration at one week defined as worse WHO ordinal status measured on the original scale, ICU admission, and 28-day MV or death. This was the cutoff value of bamlanivimab for hospitalized COVID-19, in which there was evidence of a trend toward differential treatment effect when stratified by plasma N-antigen above/below 1,000 pg/mL [13]. The sensitivity, specificity, positive predictive value (PPV), and negative predictive value (NPV) for each outcome were calculated. Separate unadjusted logistic regression models were fit, and areas under the receiver operating curve (AUROC) were estimated. We also tested the sensitivity, specificity, PPV, and NPV of internally derived cutoffs using the optimum Youden index for each outcome.

For all analyses, a two-sided *p* value of < 0.05 was considered significant. Statistical analysis was performed using Stata version 17.0 (College Station, TX).

## Results

### Baseline characteristics, viral N-antigen concentrations, and plasma biomarkers

**Fig. 1:**
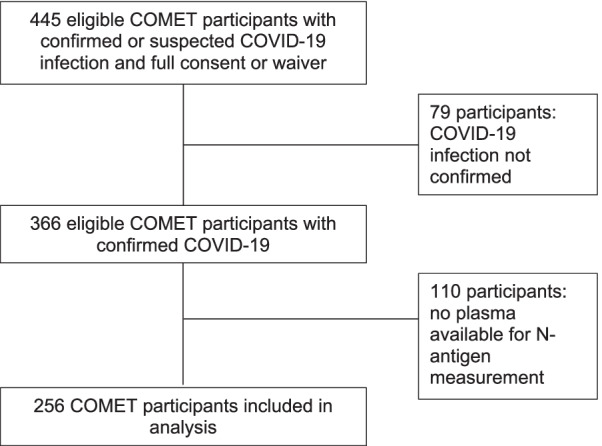
Flowchart of COMET patients included in the study

Plasma volume was sufficient for viral N-antigen measurements in 256 COVID-positive COMET subjects (Fig. 1), consecutively enrolled between March 2020 and August 2021. Baseline characteristics of study participants are presented in Table 1. 71% of participants received remdesivir, 43% received systemic steroids (for > 1 day and within a week prior to enrollment or later), 4% received tocilizumab, and < 1% received monoclonal antibody treatment at any point during hospitalization.

**Table 1:** Baseline characteristics of study participants

Age (years)	57 (16)
Sex	
Female	85 (33.2%)
Male	170 (66.8%)
Vaccinated	51 (20.0%)
Race	
White	44 (17.2%)
American Indian/Alaska Native	2 (0.8%)
Asian	45 (17.6%)
Black/African-American	22 (8.6%)
Native Hawaiian/Other Pacific Islander	8 (3.1%)
Other/Multiple, refused, or unknown	1 34 (52.3%)
Ethnicity	
Hispanic/Latino	136 (53.5%)
Not Hispanic/Latino	115 (44.9%)
Patient Refused or unknown	4 (1.6%)
BMI (kg/m^2^)	29.9 (25.4–35.4)
Baseline O_2_ saturation (*n* = 254)	95 (90–97)
Baseline WHO ordinal scale	
Hospitalized, No O_2_	46 (18.0%)
NC < 6L	93 (36.5%)
> 6L, HFNO, or NIV	57 (22.4%)
MV	10 (3.9%)
MV + organ support	49 (19.2%)
Symptom duration (days) (*n* = 236)	8 (5–10)
Cigarette smoker (*n* = 254)	
Never	161 (63.4%)
Former	57 (22.4%)
Current	21 (8.3%)
Unknown	15 (5.9%)
Vaping (*n* = 254)	
Never	172 (67.7%)
Current	2 (0.8%)
Unknown	80 (31.5%)
Hypertension (*n* = 255)	126 (49.6%)
CKD (*n* = 255)	38 (15.0%)
Diabetes (*n* = 244)	93 (38.1%)
Immunosuppression	39 (15.3%)

For variables with missing observations, the number complete is indicated in parentheses. Categorical data are presented as n (%). Normally distributed continuous data are presented as mean (SD). Non-normally distributed continuous data are presented as median (IQR)

*BMI* body mass index, *CKD* chronic kidney disease, *WHO* World Health Organization

N-antigen was present above the lower limit of detection (3 pg/mL) in 84% of participants. There was no significant difference in the percentage of participants with detectable N-antigen levels by baseline WHO ordinal scale (*p* = 0.33), though there was a trend toward a lower percent detectable in those who were intubated at D0 (78% detectable) as compared to those who were not (86% detectable). Among subjects with symptom duration recorded, median symptom duration was shorter (8 days) in participants with detectable N-antigen as compared to those without (11 days, *p* = 0.015). When limited to those with 14 days or fewer of symptoms, plasma viral antigen level was detectable in 91% of participants as compared to 57% of participants with a symptom duration of greater than 14 days.

The overall distribution of plasma N-antigen concentrations was right-skewed (Additional file 1: Fig. S1) with a median of 735 pg/mL (range 2.9–80,108; IQR 24–4574). There was no significant difference in baseline viral antigen concentration across WHO ordinal categories at D0. Baseline characteristics were compared across quartiles of viral antigen concentration (Additional file 1: Table S2). The proportion of white patients (*p* = 0.004), median symptom duration (*p* < 0.001), and baseline oxygen saturation (*p* = 0.042) declined across viral antigen quartiles, while the proportion of participants with hypertension increased (*p* = 0.043). Median viral antigen concentration was lower among males (537, IQR 15–3838 pg/mL) than females (1327, IQR 50–5115 pg/mL), but this comparison did not reach statistical significance (*p* = 0.16).

N-antigen concentration was significantly lower among current smokers (*n* = 21) than current non-smokers (*n* = 235) (43 [IQR 9–1060] pg/mL versus 804 [IQR 27–4887] pg/mL, *p* = 0.04, (Additional file 1: Fig. S2). Median symptom duration, age, BMI, and comorbidities such as hypertension and diabetes did not differ significantly between smokers and non-smokers, though there were more males (*n* = 17, 81% of smokers and 9% of males) than females (*n* = 4, 19% of smokers and 4% of females) among the smokers.

### N-antigen association with plasma proteins, cytokines, and chemokines

**Fig. 2:**
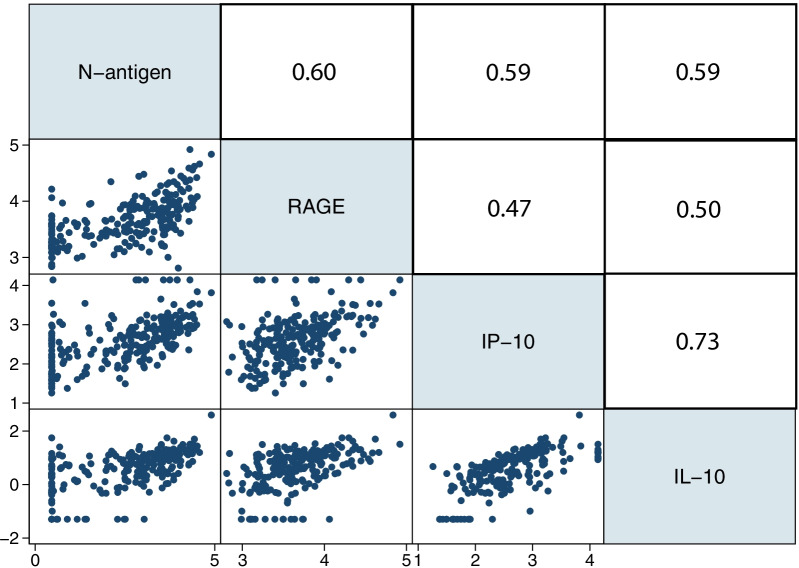
Correlation matrix for plasma biomarker and N-antigen concentrations. Biomarkers are presented on the log_10_ scale. Spearman correlation coefficients are presented above the diagonal. RAGE, *n* = 235; IL-10, *n* = 230; IP-10, *n* = 231. *P* value for all correlations = 0.01 when adjusted for multiple comparisons

Additional plasma proteins, cytokines, and chemokines from D0 samples had been previously measured for some participants. The correlations between viral N-antigen and ten selected proteins, cytokines, and chemokines at D0 were tested. RAGE, IL-10, and IP-10 were significantly correlated with viral N-antigen concentration after correction for multiple comparisons (Fig. 2). IL-10 and IP-10 were most closely correlated with one another, and RAGE demonstrated a modest correlation with IL-10 and IP-10. Other biomarkers were not significantly correlated with N-antigen concentration after correction for multiple comparisons. The distribution of all measured plasma proteins, cytokines, and chemokines across viral N-antigen quartiles is shown in Additional file 1: Table S3. There was also no correlation between plasma N-antigen levels and the earliest (D0 or D1) clinical laboratory markers of inflammation (C-reactive protein [CRP], lactate dehydrogenase [LDH], and ferritin) or dysregulated coagulation (D-dimer).

### N-antigen association with clinical outcomes

**Fig. 3:**
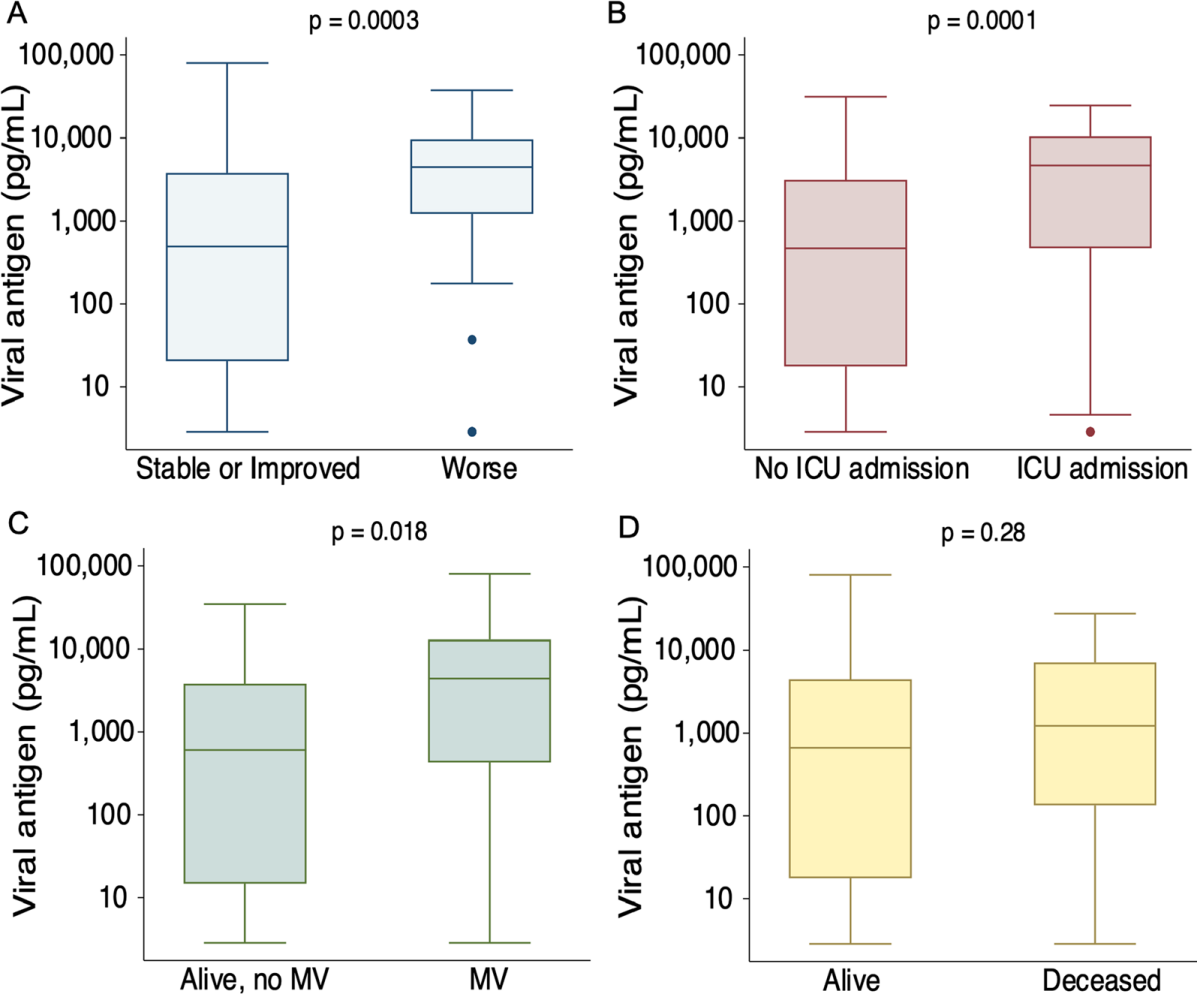
**a** Plasma SARS-CoV-2 N-antigen concentration on D0 by change in clinical status on the World Health Organization ordinal scale. **b** Plasma viral N-antigen concentration on D0 in patients who were not initially admitted to the ICU at the time of study recruitment, stratified by ICU admission during hospitalization. **c** Plasma SARS-CoV-2 N-antigen concentration on D0 stratified by mechanical ventilation at 28 days.** d** Plasma SARS-CoV-2 N-Antigen concentration on D0 stratified by death at 28 days. Plasma N-antigen concentration is presented on log_10_ scale. *P* values represent the results of the Wilcoxon rank-sum test

We next tested the association between plasma N-antigen concentration at D0 and clinical status at one week defined by the modified 5-point WHO ordinal scale (see methods). One-week outcome data were complete for 254 of 256 participants. Two participants were transferred to another hospital before one week with unknown subsequent clinical status. Plasma N-antigen concentrations were significantly higher at hospital presentation for the 35 participants whose clinical status was worse by the WHO ordinal scale one week after study enrollment compared to those who were stable or improved (4507 [IQR 1225–9665] pg/mL vs. 483 [IQR 15–3811] pg/mL, p = 0.0003, Fig. 3a). In a proportional odds model with a modified ordinal outcome (Additional file 1: Table S1), each 500 pg/mL increase in D0 plasma N-antigen concentration was significantly associated with a worse one-week outcome in both unadjusted (OR 1.05, 95% CI 1.03–1.07) and fully adjusted (OR 1.05, 95% CI 1.02–1.08) models (Table 2). One hundred and sixty-eight participants were not admitted to the ICU at the time of enrollment, of whom 40 were subsequently admitted to the ICU. We tested the association between D0 plasma N-antigen among these participants and any ICU admission during their hospitalization. The median plasma N- antigen concentration among those ever admitted to the ICU was 4697 (IQR 482–10,410) pg/mL compared to 471 (IQR 18–3142) pg/mL among the 128 never admitted to the ICU (*p* = 0.0001, Fig. 3b). Each 500 pg/mL increase in enrollment plasma N-antigen concentration was associated with an unadjusted OR of 1.08 (95% CI 1.04–1.12) and a fully adjusted OR of 1.16 (95% CI 1.07–1.25, Table 2) for ICU admission.

**Table 2:** Associations between D0 N-antigen concentration and four clinical outcomes

OR per 500 pg/mL increase (95% CI)	*p* value
*One-week ordinal outcome (n = 252)*	
Unadjusted	
1.05 (1.03–1.07)	< 0.0001
Fully adjusted	
1.05 (1.02–1.08)	< 0.0001
*ICU admission (n = 168)*	
Unadjusted	
1.08 (1.04–1.12)	< 0.0001
Fully adjusted	
1.16 (1.07–1.25)	< 0.0001
*28-day mechanical ventilation (n = 221)*	
Unadjusted	
1.04 (1.01–1.06)	0.002
Fully adjusted	
1.04 (1.00–1.08)	0.060
*28-day mortality (n = 244)*	
Unadjusted	
1.01 (0.98–1.03)	0.63
Fully adjusted	
0.98 (0.96–1.01)	0.31

Fully adjusted models include age, sex, body mass index (kg/m^2^), race, diabetes, hypertension, symptom duration (days), immunosuppression, current smoking status, remdesivir treatment, steroid treatment, baseline 8-point WHO ordinal scale, and date of enrollment. One-week and ICU models include vaccination status. ICU model excludes subjects admitted to the ICU at study enrollment. Mechanical ventilation model excludes subjects who were deceased on day 28

At 28 days, 22 participants were mechanically ventilated, 23 had died, 199 were either still hospitalized and not intubated or discharged alive and free of mechanical ventilation, and 12 had been transferred to another acute facility with unknown status at 28 days. Among participants with complete 28-day outcome data, those who were mechanically ventilated at 28 days had a median D0 N-antigen concentration of 4413 (IQR 432–12,941) pg/mL compared to 574 (14–3811) pg/mL among those who were alive and free of invasive ventilation (*p* = 0.018, Fig. 3c). Although D0 viral N-antigen concentration was numerically higher among participants who died (1225 [IQR 135–7105] pg/mL) relative to those who were alive on day 28, this difference was not statistically significant (651 [IQR 15–4404] pg/mL, *p* = 0.28, Fig. 3d).

Each 500 pg/mL increase in enrollment plasma N-antigen concentration was associated with an unadjusted OR of 1.04 (95% CI 1.01–1.06) and a fully adjusted OR of 1.03 (95% CI 1.00–1.05, Table 2) for mechanical ventilation on day 28. The unadjusted OR for death per 500 pg/mL increase in N-antigen concentration was 1.01 (95% CI 0.98–1.03), and the adjusted OR was 0.99 (0.96–1.02).

### Prognostic discrimination of high D0 plasma N-antigen

We tested the prognostic utility of an externally defined cutoff for high plasma N-antigen concentration of 1000 pg/mL or above at D0 for risk stratification. In order to generate sensitivity/specificity and areas under receiver operating curves (AUROC), the dichotomous outcome of the ordinal scale better or worse than at enrollment was used for the one-week outcome. ICU and 28-day mechanical ventilation or death outcomes were the same as in the regression models above. D0 plasma N-antigen concentration was 1000 pg/mL or greater in 46% of participants. Sensitivity, specificity, and negative and positive predictive values (NPV and PPV) of this cutoff for each clinical outcome are shown in Table 3. A comparison of the prognostic discrimination for internally derived cutoffs for each outcome by the optimum Youden index as well as the odds ratios associated with each outcome for the internally derived outcome-specific cutoffs in multivariable models can be found in the Supplemental Results (Additional file 1: Tables S4, S5). The internally derived cutoff outperformed the externally derived cutoff for the outcome of 28-day mechanical ventilation. For the other outcomes, the internally derived cutoffs only marginally improved prognostic discrimination or were not statistically different from 1000 pg/mL.

**Table 3:** Prognostic accuracy of D0 plasma N-antigen concentration ≥ 1,000 pg/mL for four clinical outcomes

	N-antigen < 1000 pg/mL	N-antigen ≥ 1000 pg/mL	Sensitivity (%)	Specificity (%)	AUROC (95% CI)	PPV (%)	NPV (%)
One-week ordinal status (*n* = 254)							
Stable/Improved	130	90	77	59	0.68 (0.60–0.76)	23	94
Worse	8	27					
ICU admission (*n* = 168)							
No ICU	79	49	70	62	0.66 (0.57–0.74)	36	87
ICU	12	28					
28-day mechanical ventilation (*n* = 220)							
Alive and free of MV	115	84	59	58	0.58 (0.47–0.69)	13	93
MV	20	25					
28-day mortality (*n* = 244)							
Alive	79	49	52	56	0.54 (0.43–0.65)	11	92
Deceased	12	28					

*AUROC* area under the receiver operating curve, *MV* mechanical ventilation, *NPV* negative predictive value, *PPV* positive predictive value

## Discussion

The results of this study support the potential value of measuring SARS-CoV-2 nucleocapsid antigen levels in the plasma as a reliable method for detecting the dissemination of viral products into the blood that has biological relevance and prognostic value for clinical outcomes. Plasma viral N-antigen concentration has several advantages over plasma viral RNA as a pathogenic and prognostic biomarker in COVID-19. Previous studies have found that SARSCoV-2 RNA is detectable approximately 10–34% of the time in pooled estimates [17, 26]; increasing the sensitivity for detection may require advanced laboratory techniques that would be difficult to apply for clinical use [39]. Although plasma RNA is associated in some studies with clinical outcomes, it is also most easily detected in patients who are severely ill [12, 18, 19, 25, 40], limiting the prognostic utility of SARS-CoV-2 RNA in patients with early or non-critical disease. By contrast, plasma N-antigen was detectable in 85% of all study participants in our study, and in 91% of those with 14 or fewer days of symptoms prior to enrollment, in line with prior findings [13, 27, 28, 41]. N-antigen was also detectable in plasma across the spectrum of disease severity with a trend toward greater detection in study participants who were not mechanically ventilated at the time of enrollment, possibly because of longer symptom duration in these patients or prior receipt of anti-viral therapies.

While plasma SARS-CoV-2 RNA concentrations are often associated with markers of inflammation and vascular damage [12, 19], we found that plasma N-antigen levels demonstrated weak or no correlation with markers of inflammation (IL-6, IL-8, sTNFR-1), coagulation (protein C), and endothelial injury (Ang-2). Similarly, N-antigen levels were not correlated with markers of inflammation and dysregulated coagulation measured as part of clinical care (LDH, ferritin, CRP, and D-dimer). However, N-antigen levels were correlated with RAGE, IL-10, and IP-10. Elevated D0 RAGE, a marker of type I pneumocyte injury [42, 43], supports recent evidence that the pathogenesis of COVID-19 is likely mediated by damage to the alveolar epithelium [44], perhaps as an early and sentinel event to subsequent endothelial injury and inflammatory response [1]. Prior studies have also identified IP-10 and IL-10 as biomarkers with more specificity for viral infection, including COVID-19, relative to other causes of respiratory failure [45]. Whether the association between N-antigen and IL-10 reflects an appropriate compensatory response to mitigate immune-mediated tissue damage [46] or an adaptive feature of SARS-CoV-2 that suppresses viral clearance by the host [47] requires further study.

In this cohort, the baseline plasma N-antigen concentrations of current smokers were significantly lower than those of non-smokers or former smokers. These differences were not explained by differences in baseline characteristics such as age or comorbidities, nor did symptom duration prior to presentation differ significantly. Smokers were male-predominant, and viral antigen concentrations were lower among males than females in our cohort, which could explain part of the observed difference. The absolute number of smokers in our cohort was small, constituting approximately 9% of the sample, which is lower than the percentage of US adult smokers but similar to San Francisco’s adult smoking prevalence [48, 49]. Nevertheless, these results are consistent with reports that smokers are at lower risk for severe COVID-19 [50].

In addition to insights into COVID-19 biology, the results of this study suggest an important role for plasma N-antigen levels in clinical prognostication. Our results indicate that early plasma N-antigen levels might contribute to the risk stratification for clinical deterioration (clinical deterioration at one week and ICU admission). The unadjusted association between viral N-antigen levels in the plasma and mechanical ventilation was statistically significant, but the fully adjusted association did not meet the threshold for statistical significance, though there was a substantial trend toward an association. Although we did not observe an association between high antigen levels and mortality, we note that our cohort was relatively young with a median age of 57 years. It is possible that an association between N-antigen concentrations and mortality would be observed in an older cohort with a higher mortality rate, or in a cohort with longer follow-up beyond 28 days. High N-antigen concentration was more sensitive than specific for adverse clinical outcomes. Importantly, low N-antigen concentration had a good negative predictive value for adverse outcomes. These findings indicate that plasma N-antigen concentration early in a patient’s hospital course could be used in addition to clinical information to identify those patients who are unlikely to progress to severe illness or death. Plasma N-antigen concentration, which can be measured within hours, could be a valuable biomarker for combined prognostic and predictive clinical trial enrichment in the context of the evidence of differential treatment response by antigen level in a randomized monoclonal antibody trial [13, 51].

Strengths of our study include prospective enrollment in a diverse observational cohort with a range of illness severity levels. In contrast to other studies of N-antigen levels in the plasma, our results are quantitative [37]. Study participants were well phenotyped both clinically and biologically. Study participants were enrolled from both a tertiary care center and a safety net hospital, and our study population received treatments reflecting the evolving standard of care during the pandemic.

Our study also has limitations. Although our results provide insight into the clinical and biological importance of circulating SARS-CoV-2 viral antigens, it does not explain why plasma N-antigen levels are elevated in patients with more severe disease. Possible mechanisms include uncontrolled viral replication in the lungs, alveolar epithelial–capillary barrier disruption, apoptosis of infected epithelial cells in the upper and lower respiratory tracts, and direct infection of circulating leukocytes [52]. Characterization of the mechanisms driving systemic viral antigen dissemination and the therapeutic implications of these mechanisms are warranted. There was no limit for symptom duration prior to enrollment, and some participants were transferred from another hospital, which could confound the relationship between enrollment plasma SARS-CoV-2 N-antigen levels and initial disease severity. Additionally, our cohort was relatively young. Representation of the delta and omicron variants and omicron subvariants is unlikely in our study population as the timeline of enrollment was before the widespread of these variants in North America. It is also possible that the approximately 5% missing 28-day outcomes data due to study participants who were transferred to another facility contributed to bias in the outcome. Participants who were transferred had higher baseline and 1-week severity of illness and were more likely to have N-antigen levels above the 1000 pg/mL cutoff relative to those patients with complete follow-up. This may have biased results of 28-day outcome data toward the null. Lastly, cutoffs for prognostic discrimination are hypothesis-generating only and require further validation in other cohorts.

## Conclusions

In summary, plasma SARS-CoV-2 N-antigen levels are readily measured in the plasma of COVID-19 patients independent of baseline disease severity. In combination with other plasma biomarkers, especially elevated levels of plasma RAGE, N-antigen levels provide important biological insight into the pathogenesis of COVID-19 pneumonia, potentially emphasizing the role of alveolar epithelial cell injury. In addition, the measurement of N-antigen levels soon after hospital presentation may improve risk stratification for clinical use and for the enrichment of clinical trials.

## Supplementary Information

**Additional file 1.** Online supplement.

## Data Availability

The datasets used and/or analyzed during the current study are available from the corresponding author on reasonable request.

## Abbreviations

Ang-2: Angiopoietin-2
AUROC: Area under the receiver operating curve
BMI: Body mass index
CKD: Chronic kidney disease
CRP: C-reactive protein
COMET: COVID-19 multi-phenotyping for effective therapies
HFNO: High flow nasal oxygen
ICU: Intensive care unit
IL: Interleukin
IMPACC: Immunophenotyping assessment in a COVID-19 cohort
IP: Interferon-gamma induced protein
LDH: Lactate dehydrogenase
NPV: Negative predictive value
PPV: Positive predictive value
RAGE: Receptor for advanced glycation end-products sTNFR-1: soluble tumor necrosis factor receptor 1
WHO: World Health Organization
MV: Invasive mechanical ventilation
