# Supplementary material for: Plasma SARS-CoV-2 nucleocapsid antigen levels are associated with progression to severe disease in hospitalized COVID-19
Source: Crit Care. 2022 Sep 14;26:278. doi: 10.1186/s13054-022-04153-3 (PMC9472195; doi:10.1186/s13054-022-04153-3)
Supplement: Supplementary file 1 — Additional file 1. Online supplement. [file 13054_2022_4153_MOESM1_ESM.docx]

**Additional file 1**

**Plasma SARS-CoV-2 nucleocapsid antigen levels are associated with progression to severe disease in hospitalized COVID-19**

**ONLINE SUPPLEMENT**

**COMET consortium members and affiliations:**

Yumiko Abe-Jones: Division of Hospital Medicine, University of California, San Francisco, California.

Alexander Beagle: Department of Medicine, University of California, San Francisco, CA, USA.

Sharvari Bhide: Division of Pulmonary and Critical Care Medicine, Department of Medicine, Zuckerberg San Francisco General Hospital and Trauma Center, University of California San Francisco

Gabriela K. Fragiadakis: Department of Medicine, Division of Rheumatology, University of California, San Francisco, CA 94143, USA, CoLabs, University of California, San Francisco, CA 94143, USA, Bakar ImmunoX Initiative, University of California, San Francisco, CA 94143, USA

Ana Gonzalez: Division of Pulmonary and Critical Care Medicine, Department of Medicine, Zuckerberg San Francisco General Hospital and Trauma Center, University of California San Francisco.

Omid Jamdar: Helen Diller Family Comprehensive Cancer Center, University of California, San Francisco, CA, USA.

Norman Jones: Core Immunology Laboratory. Division of Experimental Medicine, University of California San Francisco, California, USA.

Tasha Lea: Department of Pathology, University of California, San Francisco, CA, USA.

Carolyn Leroux: Division of Pulmonary and Critical Care Medicine, Department of Medicine, University of California San Francisco, San Francisco, California, USA.

Jeff Milush: Core Immunology Laboratory. Division of Experimental Medicine, University of California San Francisco, California, USA.

Logan Pierce: Division of Hospital Medicine, University of California, San Francisco, California.

Priya Prasad: Division of Hospital Medicine, University of California, San Francisco, California.

Sadeed Rashid: Helen Diller Family Comprehensive Cancer Center, University of California, San Francisco, CA, USA.

Nicklaus Rodriguez: Helen Diller Family Comprehensive Cancer Center, University of California, San Francisco, CA, USA.

Austin Sigman: Division of Pulmonary and Critical Care Medicine, Department of Medicine, University of California San Francisco, San Francisco, California, USA.

Luz Torres Altamirano: Helen Diller Family Comprehensive Cancer Center, University of California, San Francisco, CA, USA.

Alyssa Ward: Division of Rheumatology, Department of Medicine, University of California San Francisco, San Francisco, California, USA.

Michael Wilson: Weill Institute for Neurosciences, Department of Neurology, University of California, San Francisco.

**Supplemental Methods**

Detailed inclusion and exclusion criteria

Patients were eligible for inclusion in the COVID-19 Multi-phenotyping for Effective Therapies (COMET) study if they were aged 18 years or older, had a confirmed or suspected COVID-19 diagnosis, and were admitted to the hospital (ward or ICU) within the study eligibility period. From study inception until April 23, 2021, this eligibility window was 60 hours or less. After April 23, 2021, the eligibility window was expanded to 72 hours or less. Patients transferred from non-study hospitals were eligible for inclusion from April of 2020 to November of 2020 and after April of 2021. Between November of 2020 and April of 2021, patients transferred from outside hospitals were not eligible for inclusion. Exclusion criteria were comfort care planned at the time of admission, known pregnancy, incarceration, or declining study participation.

WHO ordinal scale

The WHO ordinal scale used for the present study is outlined in **Table S1** and ranged from 1 (ambulatory with no activity limitations or new oxygen requirement) to 8 (death). Additional organ support in WHO ordinal category 7 included vasopressor support, new renal replacement therapy, or extracorporeal membrane oxygenation. Because of low numbers of study subjects at one week in categories 2 (ambulatory with new activity limitation or oxygen requirement), 6 (mechanically ventilated without additional organ support), and 8 (death), these categories were collapsed with similar categories for the ordinal regression analyses. Specifically, ambulatory categories were collapsed, and mechanical ventilation and death categories were collapsed. For analyses using dichotomous better/worse outcomes at one week, the original scale was maintained. All analyses using WHO ordinal scale as an adjustment variable utilize the original scale.

Missing data

**Plasma biomarkers**

Plasma N-antigen concentrations below the lower limit of detection (3 pg/mL) were assigned a value of 2.9 pg/mL. Other plasma biomarkers present above the detection limit of the assay were assigned the highest reported value times the assay dilution factor, and biomarkers present below the detection limit were assigned the lowest reported value divided by the dilution factor. Otherwise, missing biomarker data (unmeasured or with coefficients of variation >20%) were not imputed.

**Clinical outcomes**

For clinical status at one week, study participants discharged to an ambulatory setting on or before one week were assigned a status of ambulatory on day seven unless readmission before day seven was recorded. Outcomes for participants who were transferred to another acute care facility were not imputed. Missing ICU admission data were not imputed. For death or mechanical ventilation at 28 days, participants who were discharged alive and free of mechanical ventilation before day 28 and not known to have died after discharge and before day 28 were assigned a status of alive and free of mechanical ventilation. The 28-day outcome for study participants who were transferred to another acute facility before day 28 were not imputed.

**Missing covariate data**

For participants with complete outcomes data, symptom duration was missing for 17 (6.7%) and diabetes diagnosis was missing for 11 (4.3%). These missing covariate data were imputed using multiple imputation by chained equations (mi impute in Stata) accounting for age, sex, white race, ethnicity, body mass index (BMI, kg/m^2^), immunosuppression, log_2_-transformed viral antigen concentration, covid vaccine status, chronic kidney disease, and enrollment WHO ordinal scale and oxygen saturation with a linear link function for symptom duration in days and a logit link function for diabetes using 100 imputations. Covariate data that was only missing for study participants who also had missing outcomes data (hypertension, smoking status, baseline oxygen saturation) were not imputed.

**Supplemental results:**

Comparison of participants with complete vs. missing 28-day outcomes data.

At both enrollment and one week, all participants with missing 28-day outcome data required at least 6 L/min supplemental oxygen above pre-morbid status, and more than half required mechanical ventilation. By comparison, more than half of participants with available 28-day outcomes required 6L/min or less of supplemental oxygen at both enrollment and one week. Enrollment N-antigen concentration was non-significantly higher for participants with missing 28-day data (2460 [IQR 17-3213] vs. 665 [IQR 24- 4678] p = 0.80). Plasma N-antigen was ≥1,000 for 67% of participants missing 28-day outcomes compared to 56% of those with complete 28-day outcomes data (p = 0.15).

Prognostic performance of internally-derived N-antigen cutoffs

The optimum N-antigen cutoff for each clinical outcome and area under the receiver operating curve (AUROC) as compared to the 1,000 pg/mL cutoff are displayed in **Table S4.**  Internally-derived, outcome-specific cutoffs were superior to the externally-derived cutoff for high N-antigen level for one week clinical deterioration and mechanical ventilation at 28 days, though the difference for one week deterioration was modest. The AUROC for the cohort-derived cutoffs were comparable to 1,000 pg/mL for ICU admission or 28 day mortality. We also tested the outcome-specific cutoffs in multivariable models adjusted for the same factors as in our primary analysis.

**Table S1**. Original and modified WHO ordinal scales

| **Original WHO Ordinal Scale** | **Description** | **N per category at one week** | **Modified Ordinal Scale** | **Description** | **N per category at one week** |
| --- | --- | --- | --- | --- | --- |
| 1 | Ambulatory, no limitations | 89 | 1 | Ambulatory | 97 |
| 2 | Ambulatory, physical limitations or new oxygen requirement | 8 |  |  |  |
| 3 | Hospitalized, no oxygen requirement | 23 | 2 | Hospitalized, no oxygen requirement | 23 |
| 4 | ≤ 6 L/min supplemental oxygen | 31 | 3 | ≤ 6 liters/min supplemental oxygen | 31 |
| 5 | >6 L/min supplemental oxygen, HFNO, or NIV | 41 | 4 | >6 L/min supplemental oxygen, HFNO, or NIV | 41 |
| 6 | Mechanically ventilated | 15 | 5 | Mechanically ventilated or deceased | 61 |
| 7 | Mechanically ventilated + other organ support | 43 |  |  |  |
| 8 | Deceased | 3 |  |  |  |

**Table S2.** Baseline characteristics by viral N-antigen quartile

|  | 2.9-24.5 pg/mL | 24.6 – 735 pg/mL | 736-4574 pg/mL | 4575-80108 pg/mL | p-value |
| --- | --- | --- | --- | --- | --- |
|  | N=64 | N=64 | N=64 | N=64 |  |
| **Age, years (SD)** | 54 (16) | 58 (15) | 57 (16) | 58 (16) | 0.50 |
| **Sex** |  |  |  |  | 0.37 |
| Female | 19 (30%) | 17 (27%) | 24 (38%) | 25 (39%) |  |
| Male | 45 (70%) | 47 (73%) | 40 (62%) | 39 (61%) |  |
| **Vaccinated** | 12 (19%) | 14 (22%) | 14 (22%) | 11 (17%) | 0.90 |
| **Race** |  |  |  |  | 0.004 |
| White | 17 (27%) | 12 (19%) | 10 (16%) | 5 (8%) |  |
| American Indian/Alaska Native | 0 (0%) | 0 (0%) | 0 (0%) | 2 (3%) |  |
| Asian | 7 (11%) | 9 (14%) | 17 (27%) | 12 (19%) |  |
| Black/African American | 4 (6%) | 7 (11%) | 6 (9%) | 5 (8%) |  |
| Native Hawaiian/Other Pacific Islander | 1 (2%) | 0 (0%) | 2 (3%) | 5 (8%) |  |
| Other/Multiple, refused, or unknown | 35 (55%) | 36 (56%) | 29 (45%) | 35 (55%) |  |
| **Ethnicity** |  |  |  |  | 0.64 |
| Hispanic/Latino | 33 (52%) | 34 (53%) | 32 (50%) | 38 (59%) |  |
| Not Hispanic/Latino | 29 (45%) | 30 (47%) | 31 (48%) | 25 (39%) |  |
| Refused or unknown | 2 (3%) | 0 (0%) | 1 (2%) | 1 (2%) |  |
| **BMI (kg/m^2^) (IQR)** | 31.0 (25.2-34.9) | 28.7 (24.9-35.2) | 29.1 (24.1-34.2) | 30.6 (27.1-36.8) | 0.24 |
| **Baseline O_2_ saturation (IQR)** | 96 (92-98) | 95 (92-98) | 95 (91-97) | 94 (88-96) | 0.042 |
| **Baseline WHO ordinal scale** |  |  |  |  | 0.31 |
| Hospitalized, No O2 | 14 (22%) | 12 (19%) | 14 (22%) | 6 (9%) |  |
| NC =<6L | 21 (33%) | 21 (31%) | 27 (44%) | 24 (38%) |  |
| >6L, HFNO, or NIV | 10 (16%) | 19 (30%) | 13 (20%) | 15 (23%) |  |
| MV | 3 (5%) | 3 (5%) | 2 (3%) | 2 (3%) |  |
| MV+ organ support | 15 (24%) | 10 (16%) | 8 (11%) | 16 (27%) |  |
| **Symptom duration (days) (n = 237)** | 10 (7-15) | 7 (4-10) | 8 (5-11) | 6 (4-8) | <0.001 |
| **Cigarette smoker** (**n = 254)** |  |  |  |  | 0.20 |
| Never | 41 (64%) | 31 (48%) | 44 (69%) | 45 (73%) |  |
| Former | 12 (19%) | 21 (33%) | 13 (20%) | 11 (17%) |  |
| Current | 8 (13%) | 7 (11%) | 3 (5%) | 3 (5%) |  |
| Unknown | 3 (5%) | 5 (8%) | 4 (6%) | 3 (5%) |  |
| **Vaping (n = 254)** |  |  |  |  | 0.68 |
| Never | 43 (67%) | 40 (62%) | 43 (67%) | 46 (75%) |  |
| Current | 1 (2%) | 1 (2%) | 0 (0%) | 0 (0%) |  |
| Unknown | 20 (32%) | 23 (36%) | 21 (33%) | 16 (25%) |  |
| **Hypertension** | 29 (46%) | 24 (38%) | 32 (50%) | 41 (64%) | 0.043 |
| **CKD** | 7 (11%) | 7 (11%) | 11 (17%) | 13 (20%) | 0.49 |
| **Diabetes (n = 244)** | 20 (34%) | 22 (36%) | 23 (38%) | 28 (44%) | 0.71 |
| **Immunosuppression** | 9 (14%) | 8 (12%) | 12 (19%) | 10 (16%) | 0.79 |
| **BMI:** body mass index  **CKD**: chronic kidney disease  **WHO:** World Health Organization  For variables with missing observations, the number complete is indicated in parentheses. Categorical data are presented as n (%) and compared by Chi-square or Fisher’s exact test. Normally distributed continuous data are presented as mean (SD) and compared by unpaired t-test. Non-normally distributed continuous data are presented as median (IQR) and compared by Wilcoxon rank sum. | | | | | |

**Table S3.** Distribution of plasma biomarkers by viral N-antigen quartile.

|  | 2.9-24.5 pg/mL | 24.6 – 735 pg/mL | 735-4574 pg/mL | 4575-80108 pg/mL |
| --- | --- | --- | --- | --- |
| RAGE (pg/mL) | 1905 (1438-3709) | 3168 (2485-5509) | 5170 (2998-7970) | 9607 (5846-19873) |
| SPD  (pg/mL) | 14580 (7890-28420) | 11705 (6742-27783) | 7696 (2433-15425) | 8793 (3191-19125) |
| IL-6  (pg/mL) | 14 (3-35) | 13 (4-33) | 11 (5-33) | 27 (9-76) |
| IL-8  (pg/mL) | 6 (3-15) | 7 (4-14) | 10 (5-17) | 12 (7-21) |
| IL-18  (pg/mL) | 375 (254-555) | 406 (244-611) | 473 (310-646) | 585 (380-783) |
| IL-10  (pg/mL) | 2 (0-5) | 5 (1-9) | 8 (3-12) | 15 (9-20) |
| IP-10  (pg/mL) | 148 (53-357) | 264 (136-554) | 478 (268-1111) | 971 (676-1730) |
| Ang-2  (pg/mL) | 2760 (1622-4943) | 1879 (1135-3134) | 2074 (1291-3053) | 2374 (1400-4547) |
| sTNFR-1 | 1903 (1153-2851) | 2067 (1558-3060) | 1844 (1468-2326) | 2473 (1678-4114) |
| Protein C  (% normal) | 93 (80-116) | 87 (71-129) | 89 (77-132) | 82 (65-112) |
| Data are presented as median (IQR).  RAGE: receptor for advanced glycation end-products  SPD: surfactant protein D  IL: interleukin  IP: interferon-gamma induced protein  Ang: angiopoietin  sTNFR: soluble tumor necrosis factor | | | | |

**Table S4.** Comparison of areas under the receiver operating curve (AUROC) for cohort-derived viral antigen cutoffs for each clinical outcome compared with 1,000 pg/mL. P-values represent the results of DeLong’s test.

| **Outcome** | **Cohort-derived cutoff** | **AUROC 1,000 pg/mL (95% CI)** | **AUROC, cohort-derived (95% CI)** | **p-value for comparison** |
| --- | --- | --- | --- | --- |
| Worse WHO scale at one week | 1,183 | 0.68 (0.60-0.76) | 0.69 (0.61-0.77) | 0.024 |
| Any ICU admission | 2,200 | 0.66 (0.57-0.74) | 0.68 (0.60-0.76) | 0.31 |
| 28-day mechanical ventilation | 4,018 | 0.58 (0.47-0.69) | 0.66 (0.55-0.77) | 0.0064 |
| 28-day mortality | 4,640 | .54 (0.43-0.65) | 0.60 (0.49-0.71) | 0.077 |

**Table S5.** Associations between D0 N-antigen cohort-derived cutoffs and four clinical outcomes. Fully adjusted models include age, sex, body mass index (kg/m^2^), race, symptom duration (days), immunosuppression, diabetes, hypertension, current smoking status, remdesivir treatment, steroid treatment, enrollment date, and baseline 8-point WHO ordinal scale. One-week and ICU models include vaccination status. ICU model excludes subjects admitted to the ICU at study enrollment. Mechanical ventilation model excludes subjects who were deceased on day 28.

| **One-week ordinal outcome (n = 252)** |  |
| --- | --- |
| **OR associated with cohort-derived cutoff (95% CI)** | **p-value** |
| **Unadjusted** |  |
| 1.87 (1.19-2.94) | 0.006 |
| **Fully adjusted** |  |
| 2.29 (1.29 – 4.07) | 0.005 |
| **ICU admission (n = 168)** |  |
| **Unadjusted** |  |
| 4.57 (2.15-9.70) | <0.0001 |
| **Fully adjusted** |  |
| 12.8 (2.93-55.7) | 0.001 |
| **28-day mechanical ventilation (n = 221)** |  |
| **Unadjusted** |  |
| 3.99 (1.62 – 9.83) | 0.003 |
| **Fully adjusted** |  |
| 5.14 (1.30-20.4) | 0.019 |
| **28-day mortality (n = 244)** |  |
| **Unadjusted** |  |
| 2.50 (1.04-6.03) | 0.042 |
| **Fully adjusted** |  |
| 1.75 (0.59 – 5.22) | 0.31 |

**Figure S1.** Viral N-antigen distribution at enrollment

**Figure S2**. N-antigen concentration by current smoking status. P-value represents the result of the Wilcoxon rank sum test.

**
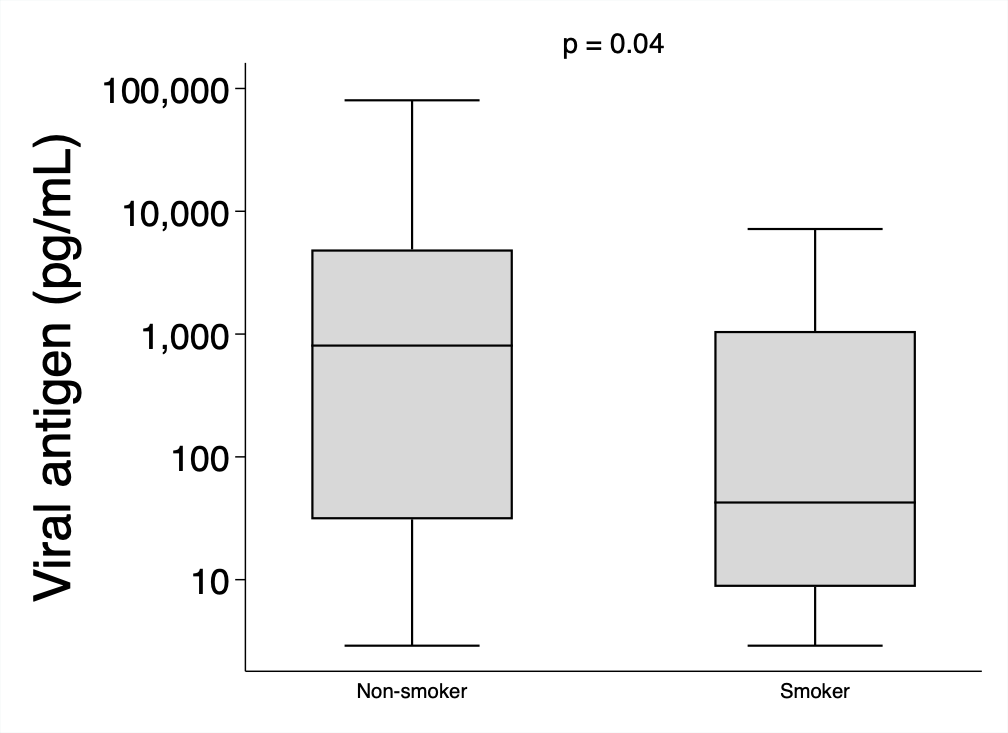
**
